# KomNET: Face Image Dataset from Various Media for Face Recognition

**DOI:** 10.1016/j.dib.2020.105677

**Published:** 2020-05-13

**Authors:** I Nyoman Gede Arya Astawa, I Ketut Gede Darma Putra, Made Sudarma, Rukmi Sari Hartati

**Affiliations:** a Department of Electrical Engineering, Politeknik Negeri Bali, Bali, Indonesia; b Information of Technology, Faculty of Engineering, Udayana University, Bali, Indonesia; c Electrical of Engineering, Faculty of Engineering, Udayana University, Bali, Indonesia; d Electrical of Engineering, Faculty of Engineering, Udayana University, Bali, Indonesia

**Keywords:** Image dataset, Face image, Face recognition, Augmentation image

## Abstract

KomNet is a face image dataset originated from three media sources which can be used to recognize faces. KomNET contains face images which were collected from three different media sources, i.e. mobile phone camera, digital camera, and media social. The collected face dataset was frontal face image or facing the camera. The face dataset originated from the three media were collected without certain conditions such as lighting, background, haircut, mustache and beard, head cover, glasses, and differences of expression. KomNet dataset were collected from 50 clusters in which each of them consisted of 24 face images. To increase the number of training data, the face images were propagated with augmentation image technique, in which ten augmentations were used such as Rotate, Flip, Gaussian Blur, Gamma Contrast, Sigmoid Contrast, Sharpen, Emboss, Histogram Equalization, Hue and Saturation, Average Blur so the face images became 240 face images per cluster. The author trained the dataset by using CNN-based transfer learning VGGface. KomNET dataset are freely available on https://data.mendeley.com/datasets/hsv83m5zbb/2.

Specifications Table

**Table:** 

Subject	Image processing, computer vision
Specific subject area	face image with three different sources
Type of data	2D-RGB image (.jpg, jpeg, png)
How data were acquired	Face images were collected from three different sources: 1. mobile phone camera 2. digital camera 3. social media (Facebook)
Data format	Raw digital image (.jpg, .jpeg, .png) Filtered augmentation image: average blur, emboss, flip, gamma contrast, gaussian blur, histogram equalization, rotate, hue and saturation, sharpen, and sigmoid contrast (.jpg, .jpeg, .png)
Parameters for data collection	the collected face images that were collected from three different sources were frontal face image or facing the camera
Description of data collection	Face images were collected from three different sources. This dataset contains the original facial image and the image that has been augmented. Face images in this collection is a frontal face which facing camera.
Data source location	Computer Laboratory, Department of Electrical Engineering, Politeknik Negeri Bali, Bali, Indonesia
Data accessibility	Dataset can be accessed on https://data.mendeley.com/datasets/hsv83m5zbb/2

## Value of the Data

• KomNET has face images originated from mobile phone camera, digital camera, and media social;
• KomNET can be used for training, validation, and algorithm comparison for face recognition.
• Dataset KomNET originated from the three media were collected without certain conditions such as lighting, background, haircut, mustache and beard, head cover, glasses, and differences of expression. The number of data training was increased by using augmentation image technique;
• KomNET can be used to develop the new CNN-based transfer learning architecture or modifying the existing architecture, e.g. Stochastic Gradient Descent or ImageNet, to improve layer efficiency on face recognition.
• Researchers who are researching about facial recognition can use this KomNET.

## 1 Data Description

KomNET dataset images contains more than 39,600 face images originated from mobile phone camera, digital camera, and media social. The purpose is training, validating and recognizing face with CNN-method, or other technique. The use of dataset for face recognition usually uses images of photos originated from single media such as dataset from mobile phone [1,2], Facebook [3], digital camera [4,5]. Algorithm development for face recognition requires images dataset from various media sources, it is a challenge for researchers because the expected results in face recognition implementation would not be an obstacle, and to date the dataset originated from these three media is not yet available.

**Fig. 1:**
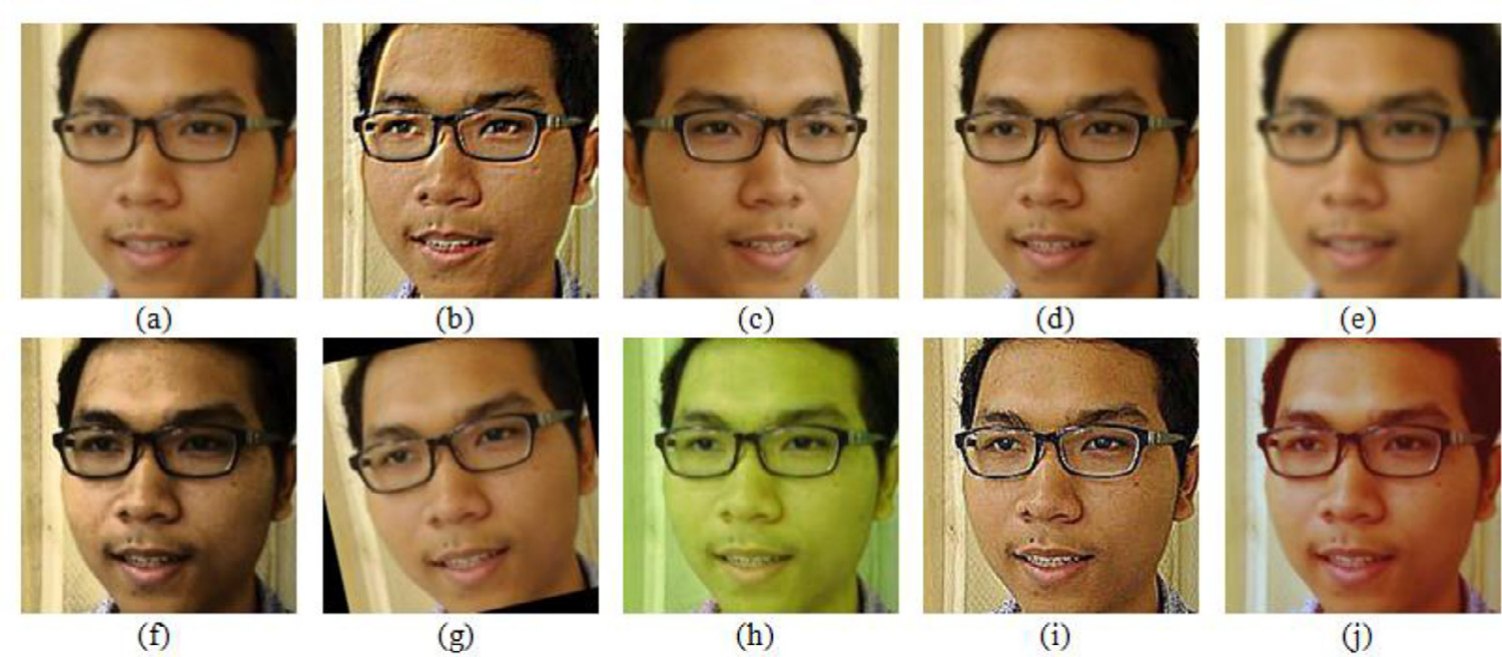
The example of image on some of augmentations (a) Average Blur, (b) Emboss, (c) Flip, (d) Gamma Contrast, (e) Gaussian Blur, (f) Histogram Equalization, (g) Rotate, (h) Hue and Saturation, (i) Sharpen, (j) Sigmoid Contrast.

Face image originated from media of mobile phone camera and digital camera were collected in Computer Laboratory, Department of Electrical Engineering, State Polytechnics of Bali. Image file or pixel sized photo was not found and the collected files were in format of .jpg or .jpeg or .png. Face images were taken with frontal face facing to the camera. Meanwhile, the other images were originated from media social of Facebook. This data collection of Facebook face images was done by collecting images from the subjects in Facebook. Every image was arranged in three main folders, namely mobile phone folder, digital camera folder, and media social folder. The most difficult thing in face recognition is the system of face recognition in different face orientation [6] such as lighting, background, haircut, mustache and beard, head cover, glasses, and differences of expression [7], e.g. smiling, laughing, being angry, and being sad. Here, the face orientation was ignored. The other obstacle in face recognition is the matching imagery is not an image originated from single device (i.e. mobile phone device and professional digital camera) [8]. Furthermore, in the dataset, there were three main folders, i.e. folder mobile phone, folder digital camera, and folder media social. In every folder, there are 4 sub folders, i.e. folder original, folder original_training, folder resize 224 × 224, and folder augmentation. Folder original is a folder containing face images from mobile phone, digital camera, and media social which are inserted into sub-sub folder with 50 cluster names with 24 face images within. Folder original_training is a folder containing images which is divided into 2 folders namely folder train containing 20 face images and folder test containing 4 face images. The following folder is folder resize 224 × 224 which contains face image that has been uniformed with size 224 × 224 pixel. The last folder is folder augmentation, it contains the augmented face images, in which there are 10 kinds of image augmentation, i.e. average blur, emboss, flip, gamma contrast, gaussian blur, histogram equalization, rotate, hue and saturation, sharpen, and sigmoid contrast. The total of dataset KomNET can be seen as the following Table 1. As we can see, Table 1 shows the number of datasets contained in KomNET. This dataset was collected from three different sources, with each number of images from each device totaling 1200 face images. Each face image is augmented with 10 types of augmentation so that it reaches 12000 face images and a total of 39600 face images. The dataset contained the original image that has not been augmented. The image has various dimensions. In addition, there are also augmented images with dimensions of 224 × 224 pixels. This image dataset uses * .jpg, * .jpeg, and * .png formats. The metadata about the dataset can be seen in Table 2. Examples of facial images after augmentation can be seen in Fig. 1. There are 10 augmentation, namely: (a) Average Blur, (b) Emboss, (c) Flip, (d) Gamma Contrast, (e) Gaussian Blur, (f) Histogram Equalization, (g) Rotate, (h) Hue and Saturation, (i) Sharpen, (j) Sigmoid Contrast. The augmentation process doubles the facial images from three different sources. Thus, KomNET's face image can be used by researchers for face recognition research.

**Table 1:** KomNET dataset face images.

Image source	Number of original images	Number of augmentation images	Total
Mobile phone	1,200	12,000	13,200
Digital Camera	1,200	12,000	13,200
Social Media	1,200	12,000	13,200
**Total**	**3,600**	**36,000**	**39,600**

**Table 2:** Metadata file.

	Original data	Augmented
File extension	*.jpg,*.jpeg,*.png	*.jpg,*.jpeg,*.png
Dimension	Various	224 × 224 pixels

## 2 Experimental Design, Materials, and Methods

KomNET images were collected from three various sources, i.e. mobile phone camera, digital camera, and media social. The collection of face images was collected with frontal face which facing camera without considering background, lighting, expression, glasses, head cover, etc. Every collected image from the three sources and image training process was separated into folder train and folder test. The collected images from these three sources have different sizes, therefore there was re-sizing process to make the size the same of 224 × 224 pixel. For good result in face recognition, there should be lots of training, and if the data is few so more data is needed and there will be minor changes in dataset. The change can be done by changing the face image such as translation, rotate, or viewpoint, size or illumination or the combination and this way can be done with image augmentation technique. The used augmentation images on dataset KomNET were average blur, emboss, flip, gamma contrast, gaussian blur, histogram equalization, rotate, hue and saturation, sharpen, and sigmoid contrast. After augmentation, the images were inserted into folder augmentation. The example of image after augmentation is presented as the following Fig. 1.

This dataset has also been used in face recognition using the CNN algorithm. Image from social media has received approval from the owner of the relevant social media account. Dataset testing was done in Computer Laboratory, Department of Electrical Engineering, Politeknik Negeri Bali, Bali, Indonesia. For the initial process, the author used wavelet method to get face feature. Furthermore, the face feature was processed by CNN-based transfer learning VGG face. The researcher [9] said that first layer feature is general and the last layer feature is specific so for the last 2 layer there was no training, only the result of feature was taken as the output. Next, this feature was processed or done by fine tuning with classification model.

Transfer learning is a good method in computer vision because it is so accurate and saving time in building a model [10]. It is because transfer learning can be used to solve similar problem without starting learning process from the beginning, by improving previous learning. Researcher [11] said that picture classification problem on deep learning can be solved through good transfer learning.

Transfer learning in computer vision is usually expressed through pre-trained model. Model pre-trained is a model usually used for training big dataset in solving similar problem. Therefore, by computation consideration for these model training, usually researchers import and use model from published literature (e.g. VGG, Inception, MobileNet). The author used pre-trained model which was based on convolutional neural networks (CNN) as conducted by researcher [12].

## Appendix Supplementary materials

Image, application 1

Image, application 2
