# Supplementary material for: KomNET: Face Image Dataset from Various Media for Face Recognition
Source: Data Brief. 2020 May 13;31:105677. doi: 10.1016/j.dib.2020.105677 (PMC7243044; doi:10.1016/j.dib.2020.105677)
Supplement: Supplementary file 2 [file mmc2.docx]

**Ethical Requirements**

According to Facebook Terms of Service in point 3.3.1, users who share or upload images have intellectual property rights. Users are free to share content with anyone. The owner of image data that we use has approved the use of image data for research purposes. We collected the approval from every user who owns the image that we used. Then, here we provide the ethical requirement document.

**
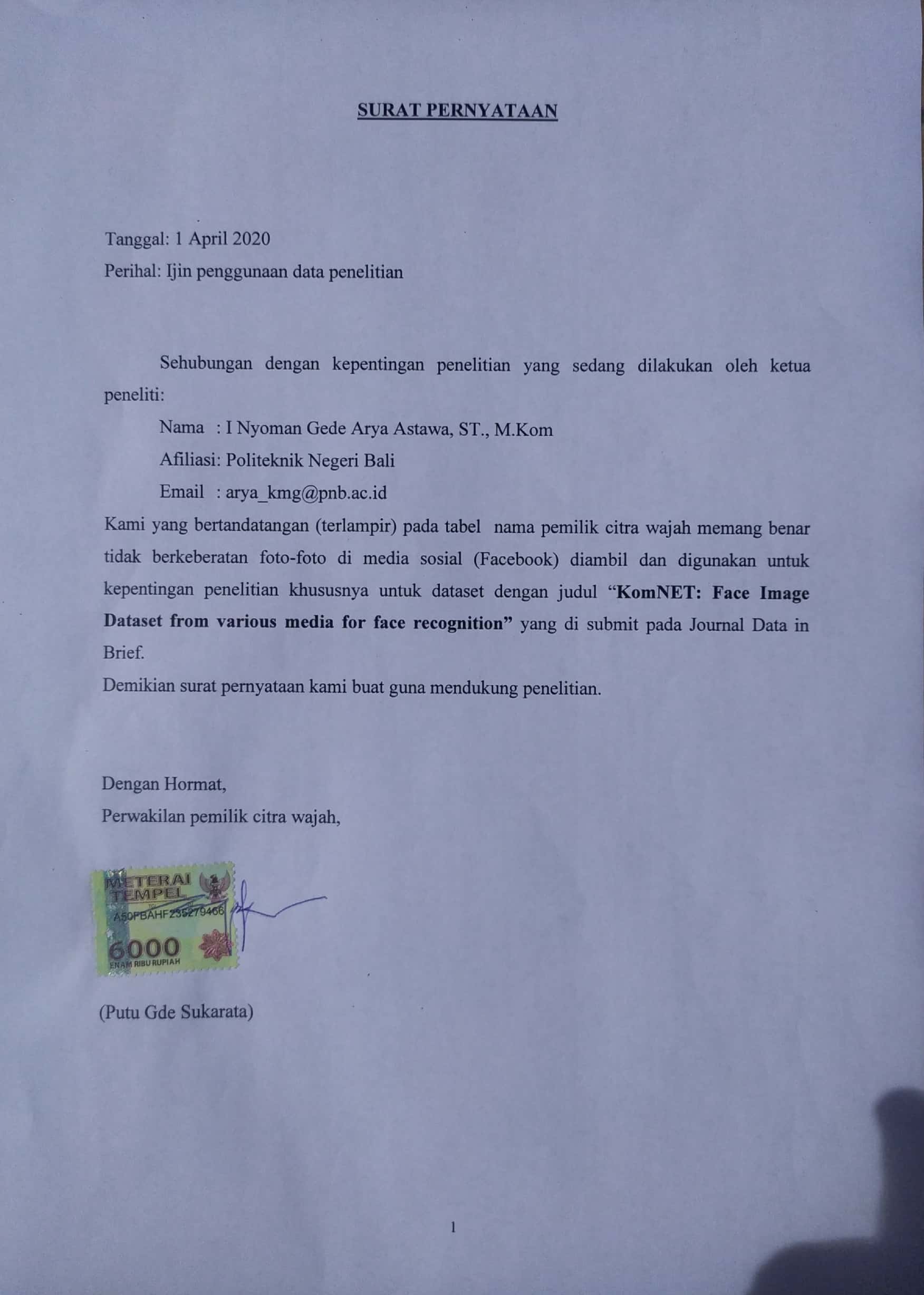
**

**
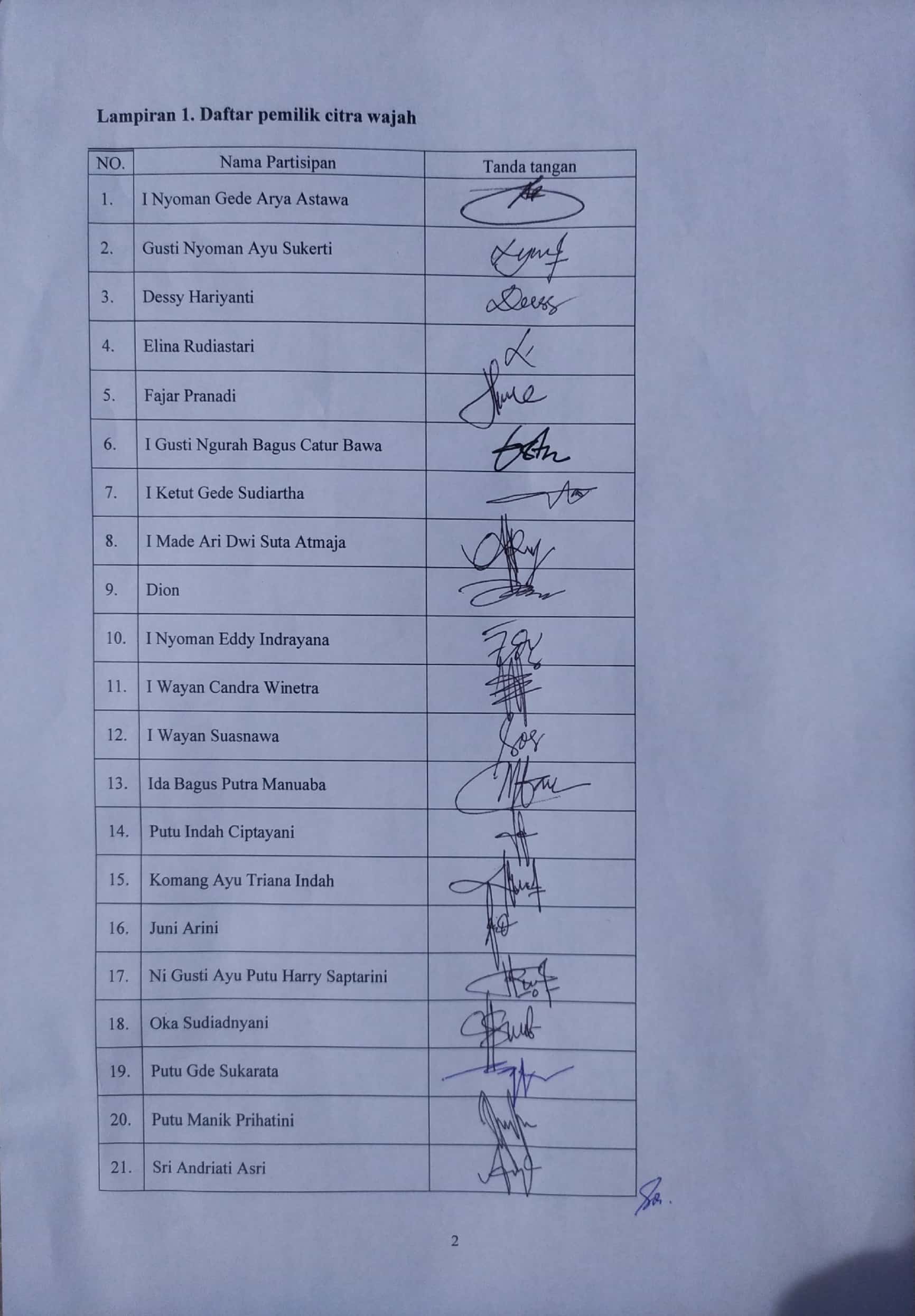
**

**
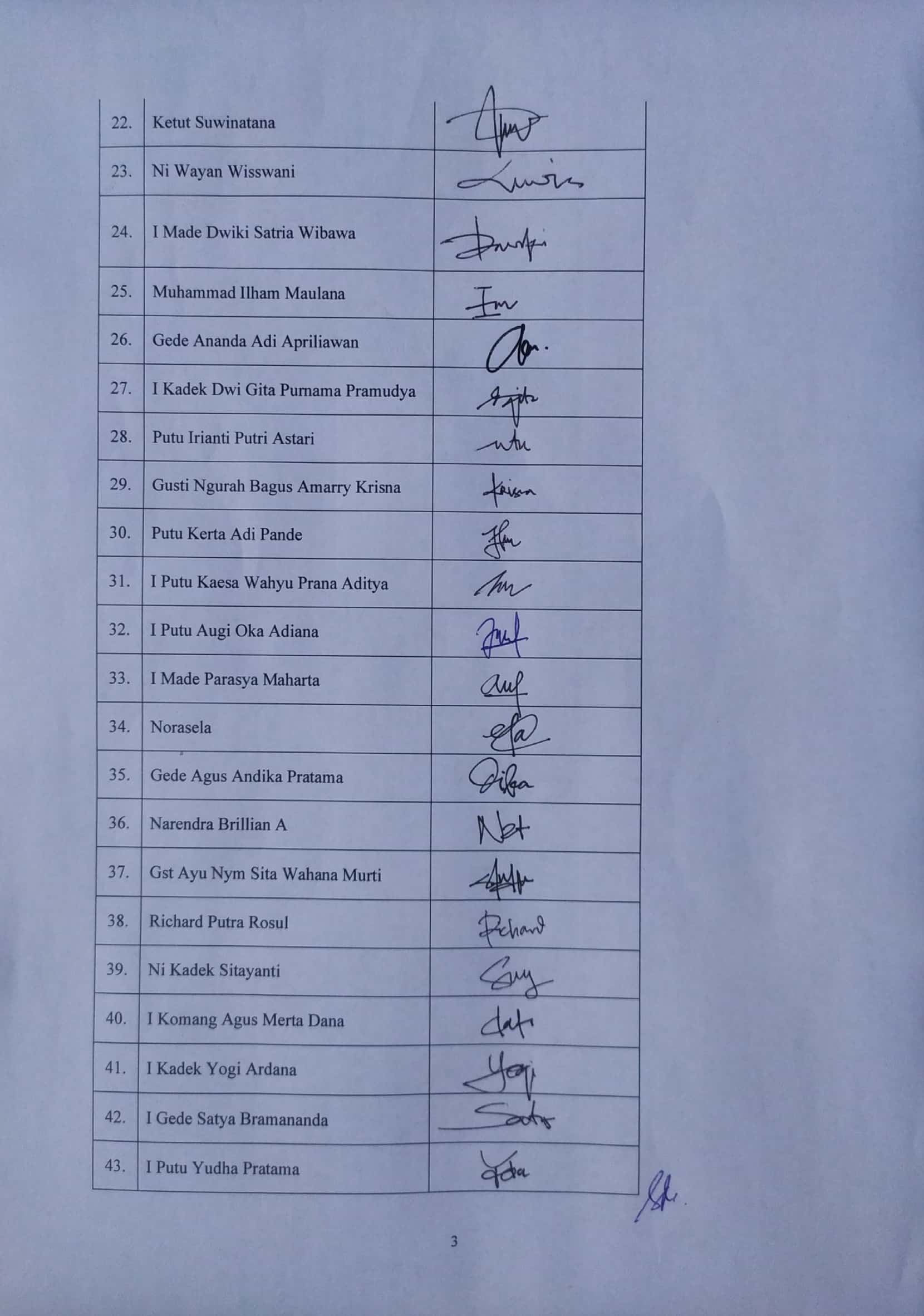
**

**
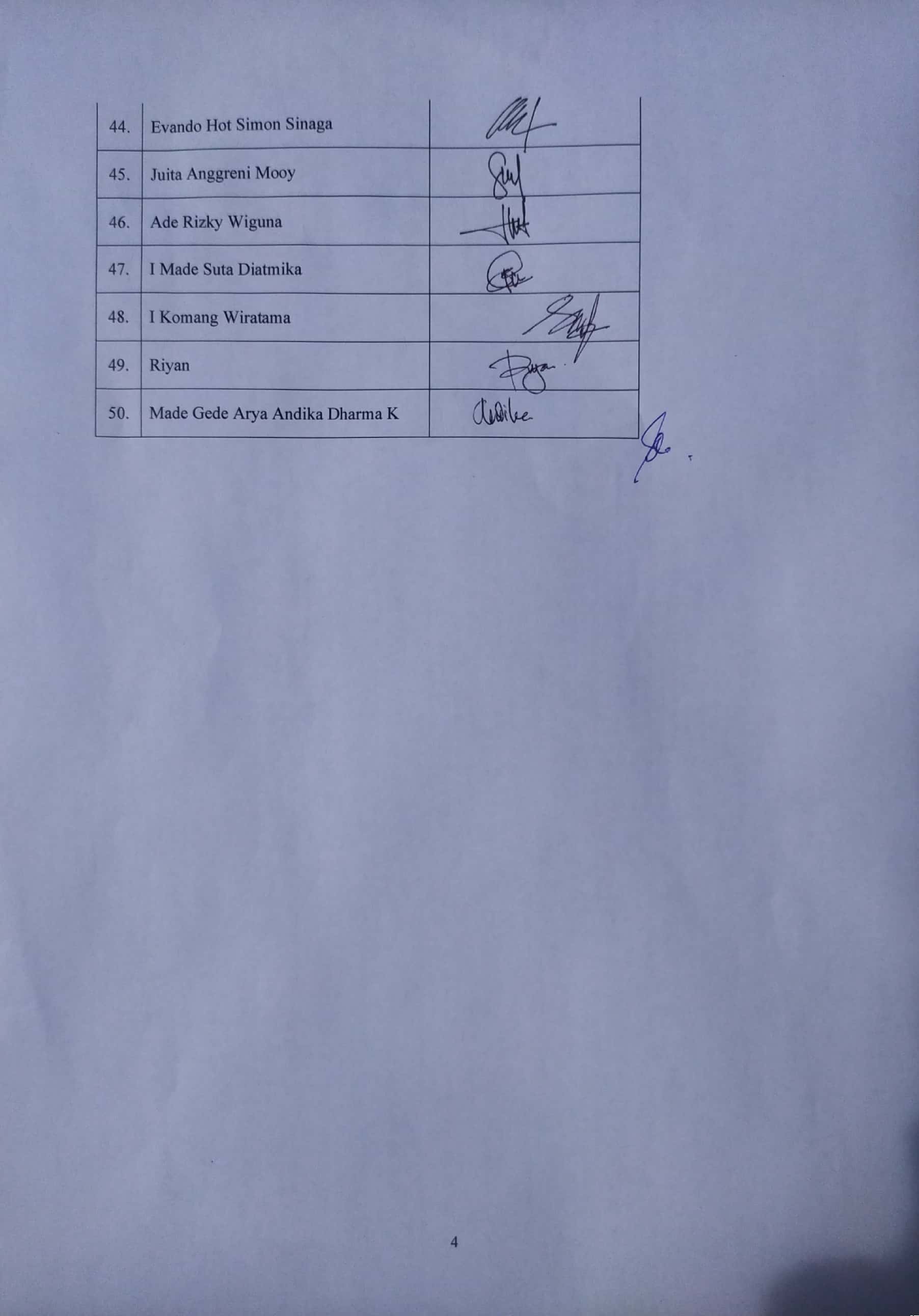
**
